# Theoretical and Experimental Studies of Phosphonium Ionic Liquids as Potential Antibacterials of MDR *Acinetobacter baumannii*

**DOI:** 10.3390/antibiotics11040491

**Published:** 2022-04-06

**Authors:** Larysa O. Metelytsia, Diana M. Hodyna, Ivan V. Semenyuta, Vasyl V. Kovalishyn, Sergiy P. Rogalsky, Kateryna Yu Derevianko, Volodymyr S. Brovarets, Igor V. Tetko

**Affiliations:** 1 V.P. Kukhar Institute of Bioorganic Chemistry and Petrochemistry, National Academy of Science of Ukraine, 1 Murmanska Street, 02094 Kyiv, Ukraine; metelitsa@bpci.kiev.ua (L.O.M.); dianahodyna@gmail.com (D.M.H.); ivan@bpci.kiev.ua (I.V.S.); vkovalishyn@bpci.kiev.ua (V.V.K.); sergey.rogalsky@gmail.com (S.P.R.); katerinaderevianko@gmail.com (K.Y.D.); brovarets@bpci.kiev.ua (V.S.B.); 2 Institute of Structural Biology, Molecular Targets and Therapeutics Center, Helmholtz Munich-Deutsches Forschungszentrum für Gesundheit und Umwelt (GmbH), 85764 Neuherberg, Germany; 3 BIGCHEM GmbH, Valerystr. 49, 85716 Unterschleißheim, Germany

**Keywords:** phosphonium ionic liquids, antibacterial, *Acinetobacter baumannii*, antioxidants, OCHEM, QSAR

## Abstract

A previously developed model to predict antibacterial activity of ionic liquids against a resistant *A. baumannii* strain was used to assess activity of phosphonium ionic liquids. Their antioxidant potential was additionally evaluated with newly developed models, which were based on public data. The accuracy of the models was rigorously evaluated using cross-validation as well as test set prediction. Six alkyl triphenylphosphonium and alkyl tributylphosphonium bromides with the C_8_, C_10_, and C_12_ alkyl chain length were synthesized and tested in vitro. Experimental studies confirmed their activity against *A. baumannii* as well as showed pronounced antioxidant properties. These results suggest that phosphonium ionic liquids could be promising lead structures against *A. baumannii*.

## 1. Introduction

*Acinetobacter baumannii* is an important nosocomial pathogen that is responsible for a wide range of human infections, resulting in high levels of morbidity and mortality. It is known that newly identified *A. baumannii* strains are resistant to most known antibiotics and able to survive for a prolonged period of time in a hospital setting, enhancing the ability to spread rapidly in the hospital which could result in the death of infected patients unless treated with nonresistant antibiotics [1,2,3,4]. The rapid global emergence of new antibiotic-resistant strains of *A. baumannii* demonstrates the high potential of this microorganism to respond quickly to environment changes. Therefore, creation of new effective antibacterial drugs is the most important task of modern drug development [5].

Long-chain ionic liquids (ILs) comprised of 1,3-dialkylimidazolium, 1-alkylpyridinium, and N-alkyl guanidinium cations are considered extremely promising antimicrobial agents since they possess broad-spectrum activity against both Gram-positive and Gram-negative bacteria including multidrug-resistant (MDR) clinical isolates [6,7,8,9,10], antifungal activity [11,12], as well as strong antibiofilm activity against a panel of pathogenic microorganisms. The length of the aliphatic chain of IL cations is known to play a crucial role towards its biological activity. Among the homologs investigated, cationic surfactants with an alkyl chain length of 12 and 14 carbon atoms have shown the highest efficiency as antimicrobial agents [6,7,8,13,14]. It is worth noting that ILs comprised of nitrogen containing heterocyclic cations are the most commonly studied, whereas the phosphorus-containing ones have been studied to a lesser extent. However, phosphonium ILs occupy a special niche among promising biocides since they have some advantages over nitrogen-containing ILs such as higher thermal stability, as well as faster kinetics of their synthesis [14]. Moreover, long-chain tetraalkyl phosphonium salts were found to have broad-spectrum antimicrobial activity [15,16,17,18,19], as well as pronounced antitumor activity [20]. In general, these compounds demonstrate higher antibacterial and antitumor activity, as well as less cytotoxicity as compared to their nitrogen-containing counterparts [15,20,21]. An important feature of tetraalkyl phosphonium-based ILs is that their activity does not have a pronounced dependence on the length of alkyl radicals bound to cations [20,22,23,24]. For example, short-chain ILs comprised of tetrabutylphosphonium cations demonstrated high activity against sixty human tumor cell lines, which was similar to that of long-chain phosphonium salts [20]. Tributyl(2-hydroxyethyl)phosphonium docusate was found to exhibit antimicrobial and antibiofilm-forming activity to several antibiotic-resistant bacteria [22,25]. Another example is tetrakis(hydroxymethyl)phosphonium sulfate, a broad-spectrum biocide targeted at a wide range of bacteria, especially sulfate-reducing bacteria [23].

Besides tetraalkyl phosphonium ILs, quaternary salts comprised of alkyl triphenylphosphonium cations are also of great interest since these compounds may have a specific biological activity. Thus, long-chain dodecyl(triphenyl)phosphonium ILs were found to induce expression of the plasma membrane of pleiotropic drug-resistant transporters in yeasts [24,25]. Mitochondria-targeted antioxidants (MTAs) are widely used in experiments for evaluating the impact of mitochondria on different pathological processes involving oxidative stress. These involve a wide range of compounds which have an antioxidant group linked to a mitochondria-targeted moiety, such as triphenylphosphonium cations [26]. It was found that SkQ1, a decyl(triphenyl)phosphonium cation conjugated to a quinone moiety, exhibited a strong antibacterial activity towards Gram-positive *Bacillus subtilis*, *Mycobacterium* sp., and *Staphylococcus aureus* and Gram-negative *Photobacterium phosphoreum* and *Rhodobacter sphaeroides* in sub micromolar and micromolar concentrations [26]. However, SkQ1 exhibited less antibiotic activity towards *Escherichia coli* due to the presence of the highly effective multidrug-resistant efflux pump AcrAB–TolC which promotes the expulsion of a wide range of molecules out of cells [26].

Surprisingly, long-chain tetraalkyl phosphonium ILs as well as alkyl triphenylphosphonium ILs have not yet been studied for their activity against multidrug-resistant clinical isolate *A. baumannii*. Only in one recent study, newly synthesized 1,3-oxazolyl triphenylphosphonium salts were found to be active against *A. baumannii* [27]. However, the presence of a biologically active heterocyclic compound 1,3-oxazole in these compounds does not allow us to fully determine whether their activity was due to this moiety or to phosphonium cations. Therefore, we decided to explore the efficacy of phosphonium ILs comprised of inert alkyl and aryl radicals against *A. baumannii*. The ability of *A. baumannii* to form biofilms contributes to its survival in adverse environmental conditions including hospital environments and medical devices [28,29]. From this point of view, the evaluation of the antioxidant activity of phosphonium salts also seems relevant. Reactive oxygen species (ROS), such as hydrogen peroxide, superoxide, and hydroxyl radicals are known to serve as regulatory signals for many bacteria to maintain a healthy redox cycle and promote microbial attachment, consequently leading to the development of biofilms [30]. A disturbance in the redox cycle can lead to oxidative stress due to the accumulation of ROS. Oxidative stress in microorganisms was found to play an important role in the regulation of redox defense mechanisms, the production of the extracellular polymer substance (EPS) matrix, and biofilm heterogeneity [31]. Therefore, compounds that target oxidative stress regulators, such as antioxidants, could potentially be exploited as a novel strategy for biofilm control. To our knowledge, there are no systematic studies of the antioxidant activity of phosphonium salts. Only tetrakis(hydroxymethyl)phosphonium chloride (THPC) was reported as an efficient antioxidant due to its rapid oxygen-scavenging ability [32,33]. The properties of THPC are due to the presence of a weak P–CH_2_OH linkage which is readily cleaved in water solutions. The formed tris(hydroxymethyl)phosphine is a fairly strong reducing agent which can scavenge oxygen to produce tris(hydroxymethyl)phosphine oxide [33]. However, the question still remains as to the antioxidant activity of nonfunctionalized phosphonium salts comprised of inert alkyl or aryl radicals.

Thus, the aim of this research was to evaluate the efficacy of alkyl tributylphosphonium and alkyl triphenylphosphonium ILs with different alkyl chain lengths as antibacterial agents against *A. baumannii* as well as their antioxidant activity in in silico and in vitro studies.

## 2. Materials and Methods

### 2.1. QSAR Modeling of Antioxidant Activity

#### 2.1.1. Dataset

The analysis was carried out using compounds with antioxidant activity obtained from the ChEMBL database and uploaded into OCHEM [34]. The structure of these compounds, their antioxidant activity, and literature sources are freely available on the OCHEM website.

The initial dataset of 1246 compounds consisted of a diverse chemical series with IC_50_ values of the molecules ranging from 0.0215 to 991 µM. The IC_50_ value determines the concentration of the sample required to inhibit 50% of the radicals. The IC_50_ values were converted into the −log(IC_50_) values and were used as the target variable to develop regression models. From the initial dataset, 20% of the compounds were randomly selected using OCHEM to form an external independent test set while the remaining molecules were used as the training set.

#### 2.1.2. QSAR

QSAR models were created with three machine learning methods, including transformer convolutional neural network (CNN) [35], transformer convolutional neural fingerprint (CNF) [36], and random forest regression (RF) [37]. The RF models were developed using three descriptor sets including E-state indices [38], ALogPS [39,40], and CDK 2.7.1 [41], which were frequently top-performing descriptors, according to our previous studies. We used the optimized parameters setting of each machine learning method provided by the OCHEM platform.

*Transformer convolutional neural network (transformer CNN).* This method uses the internal latent representation of molecules based on their SMILES notation to extract information about the molecular structure [35]. The method predicts the target value based on the average individual prediction for a batch of augmented (noncanonical) SMILES belonging to the same molecule. The deviation within the predictions for augmented SMILES can be used to determine the uncertainty of predictions.

*Transformer convolutional neural fingerprint (transformer CNF)* is similar to transformer CNN, but instead of a convolutional neural network, it uses convolutional neural fingerprint for processing the latent representation of the neural network. Like transformer CNN, it uses the augmentation technique, which was originally proposed in computer vision and was recently introduced to QSAR studies [36,42].

*Random forest (RF)* consists of many individual trees devised to operate quickly over large datasets. RF is not heavily affected by correlated descriptors since it uses random samples to build each tree in the forest. The final prediction was the average of the individual trees [37].

##### Descriptors

*E-state indices.* The calculation of electrotopological state indices is based on the chemical graph theory. E-state indices are 2D descriptors that combine both electronic and topological characteristics of the analyzed compounds [38].

*ALogPS*. The program calculates two 2D descriptors, namely the 1-octanol–water partition coefficient [39] and aqueous solubility [40].

*CDK 2.7.1.* The Chemistry Development Kit (CDK) is a set of Java module libraries for processing chemical information. CDK 2.7.1 calculates 256 molecular descriptors such as geometrical, topological, constitutional, electronic, and hybrid descriptors [41].

More details about the descriptors can be found elsewhere [34]. Pearson’s pairwise correlation method had been used for filtering each descriptor set before they were used as input for the machine learning methods. Additionally, unsupervised forward selection [43] was applied to select a representative nonredundant set of descriptors.

A fivefold cross-validation method was used to evaluate the accuracy and robustness of the QSAR models. To avoid incorrect estimation of the models due to overfitting by variable selection, OCHEM repeats all model development steps within each validation fold. In addition, we also used the test set to further confirm the quality of the developed models [44].

We used two criteria to assess the goodness of fit: the coefficient of determination *q^2^* and the mean absolute error (MAE) [34]. OCHEM also supports estimation of the applicability domain [45] of the developed machine learning models and the accuracy of predictions. Detailed descriptions of the machine learning methods, selected descriptors, statistical coefficients, and detailed validation procedures are provided in the OCHEM manual [46].

### 2.2. Biology

#### 2.2.1. Antibacterial Activity Evaluation

The MDR *A. baumannii* isolate was received from the Museum of Microbial Culture Collection of the Shupyk National Healthcare University of Ukraine collected at a Ukrainian hospital.

The antibacterial properties of PILs with high predicted activity were estimated against MDR (ampicillin-, oxacillin-, and ceftriaxone-resistant clinical isolate) *A. baumannii* by means of the disc diffusion method in a Mueller–Hinton agar [47]. The inoculum concentration of 1 × 10^5^ colony-forming units (CFU) per mL was established using a 0.5 McFarland turbidity standard and a subsequent dilution of 0.02 mL of the tested compounds was applied on standard paper disks (6 mm) which were placed on the agar plate. The compound content on a disk was 0.25, 0.5, and 1.0 µM.

The antibacterial activity of the tested compounds was identified by measuring the zone diameter of growth inhibition, which indicated the degree of susceptibility or resistance of the *A. baumannii* isolate to the test compounds. The compounds which formed zones of bacterial growth inhibition > 15 mm could be considered active.

Furthermore, the antibacterial activity of the ILs was assessed with a 96-well plate microdilution assay, a technique adapted from the methodology described by Eloff et al. [48]. The bacterial inoculum was obtained by incubating microorganisms in a Mueller–Hinton broth for 24 h, adjusting it to a final concentration of 1 × 10^5^ CFU per ml. The ILs were solubilized in sterile water, and the final concentrations for each IL were as follows: 0.4 µM, 0.78 µM, 1.56 µM, 3.12 µM, 6.25 µM, 12.5 µM, 25.0 µM, 50.0 µM, 100.0 µM, and 200.0 µM. Each plate included bacterial growth (positive control) and culture medium (negative control) for 24 h at 37 °C. The minimum concentrations without visible growth (using a binocular microscope) were defined as the concentrations which completely inhibited bacterial growth. Ofloxacin was used as a positive control in the MIC assay (0.015 µmol).

#### 2.2.2. Antioxidant Activity Evaluation

The evaluation of antioxidant properties of the PILs (10 µM) was studied in vitro using the inhibition value of the rate of ascorbate-dependent radical lipid peroxidation and represented by the malonic dialdehyde (MDA) content using the reaction with thiobarbituric acid [49]. The amount of MDA was determined photometrically at 532 nm [50]. The intensity of lipid peroxidation processes was determined by the difference between the MDA content in the experimental and control samples (solvent) and presented as the percentage of control.

## 3. Results

### 3.1. Regression Model to Predict the Antioxidant Activity

The initial dataset of 1246 compounds with antioxidant activity collected from literature was split by chance into the training (997) and test (249) sets as described in the Materials and Methods.

In the preliminary stage of the analysis, all molecules were processed by the OCHEM software [34]; 2D coordinates of atoms were recalculated, counter ions and salts were removed from molecular structures, and molecular structures were neutralized and mesomerized.

The regression models were built using several methods, as described in the Materials and Methods section. The best-performing methods (see Table 1) included those based on representation learning (transformer CNN [35] and transformer CNF [36]) as well as traditional descriptors (random forest, RF [37]). For the latter method, we selected the E-state [38], ALogPS [39], and CDK 2.7.1 [41] descriptors, which contributed to the top-performing model for the RF.

The coefficient of determination *q*^2^ values of the developed models were 0.73–0.77 and 0.72–0.77 for the training and test sets, respectively. Thus, the models demonstrated high accuracy and robustness for prediction of the antioxidant activity. Other statistical parameters of the models were summarized in Table 1 as well as in the Supplementary Materials (Figure S2). A consensus model, which was an average of three individual models, was used to provide a quantitative evaluation of potential antioxidant agents as described in the next section. Moreover, the standard deviation of these individual models was used to estimate the applicability domain of the consensus model [45].

**Table 1:** Statistical coefficients of the regression models to predict antioxidant activity.

N	Method	Training Set ^a^		Test Set ^a^	
		*q* ^2^	*MAE* ^c^	*q* ^2^	*MAE*
1	Transformer CNN	0.77 ± 0.02	0.3 ± 0.01	0.75 ± 0.05	0.28 ± 0.02
2	Transformer CNF	0.76 ± 0.02	0.3 ± 0.01	0.77 ± 0.04	0.28 ± 0.02
3	RF	0.73 ± 0.02	0.32 ± 0.01	0.72 ± 0.04	0.3 ± 0.02
4	*Consensus* ^b^	0.77 ± 0.02	0.29 ± 0.01	0.77 ± 0.04	0.27 ± 0.02

^a^ The training and test datasets included 997 and 249 molecules, respectively. ^b^ The consensus model was the average of the transformer CNN, transformer CNF, and RF models. ^c^ MAE is the mean absolute error and *q*^2^ is the coefficient of determination, respectively. The RF model was based on the E-state [38], ALogPS [39] and CDK 2.7.1 [41] descriptors.

### 3.2. Selection of Compounds with the Help of In Silico Tools

A virtual set of ILs was generated as a combinatorial library based on the recommendations of an experienced synthetic organic chemist (who suggested which types of modifications of PILs could be synthesized). It included eleven compounds with different substitution patterns (see the Supplementary Materials). All the compounds were run through the previously published consensus classification model [27]. Six compounds predicted as active among those with the most confident predictions (>60%) were selected (Table S1). The compounds were analyzed using a set of structural alerts [51] implemented in OCHEM to screen and exclude compounds with potential toxicity. All the selected compounds passed these filters. These compounds were then tested using a consensus antioxidant model developed in the previous section. All the compounds were predicted as moderately antioxidant active compounds [52] (see Table S2). Therefore, all the six compounds were retained for synthesis and experimental testing (Table 2 and Table S1). The results of biological testing of the newly synthesized compounds confirmed the theoretical predictions of their activities (Table 2, Table 3 and Table 4) as described in the following sections.

### 3.3. Synthesis of PILs

Below, we provide a description of the synthetic protocols used to synthesize the analyzed compounds.

#### 3.3.1. Initial Materials and Structure Confirmation

The following chemicals were used for the synthesis of ionic liquids: triphenylphosphine (95%), tributylphosphine (93.5%), 1-bromododecane (97%), 1-bromodecane (98%), 1-bromooctane (99%), acetonitrile (99.5%), ethyl acetate (99.7%), and hexane (95%) (Sigma-Aldrich, St. Louis, MO, USA). ^1^H NMR spectra were recorded in CDCl_3_ and DMSO-d_6_ on a Varian Gemini-2000 (400 MHz) spectrometer using TMS (tetramethylsilane) as the internal standard.

#### 3.3.2. Synthesis of Ionic Liquids

**Scheme 1:**
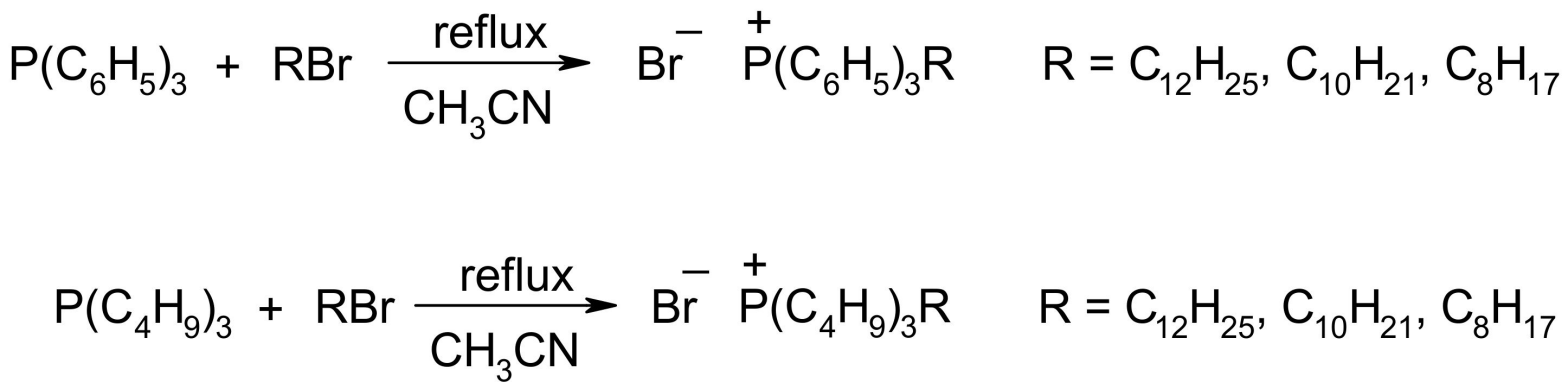
Synthesis of phosphonium ILs (see also Supplementary Table S3).

Long-chain alkyl triphenylphosphonium (1–3) and alkyl tributylphosphonium (4,5) ILs were synthesized according to Scheme 1. To the stirred solution of triphenylphosphine or tributylphosphine (0.05 mol) in 50 mL of acetonitrile, we added bromoalkane (0.06 mol), and the mixture was heated to reflux for 24 h. Acetonitrile was distilled, the product was washed with an ethyl acetate–hexane mixture (1:3 *v*/*v*, 3 × 100 mL). The residual solvents were removed in vacuum (10 mbar) at 60 °C.

1. Octyl(triphenyl)phosphonium bromide (PPh_3_C_8_–Br), CASRN 42036-78-2.
  Colorless solid, m.p.: 61–63 °C.
  ^1^H NMR (400 MHz, DMSO-d_6_): σ = 0.81 (t, 3H, CH_3_), 1.19 (m, 8H, CH_2_), 1.48 (m, 4H, PCH_2_CH_2_CH_2_CH_2_), 3.56 (m, 2H, PCH_2_), 7.76–7.84 (m, 15H, Ar–H).
2. Decyl(triphenyl)phosphonium bromide (PPh_3_C_10_–Br), CASRN 32339-43-8.
  White solid, m.p.: 86–88 °C.
  ^1^H NMR (400 MHz, DMSO-d_6_): σ = 0.83 (t, 3H, CH_3_), 1.2 (m, 12H, CH_2_), 1.46 (m, 4H, PCH_2_CH_2_CH*_2_*CH_2_), 3.6 (m, 2H, PCH_2_), 7.8–7.9 (m, 15H, Ar–H).
3. Dodecyl(triphenyl)phosphonium bromide (PPh_3_C_12_–Br), CASRN 15510-55-1.
  White solid, m.p.: 89–92 °C.
  ^1^H NMR (400 MHz, DMSO-d_6_): σ = 0.83 (t, 3H, CH_3_), 1.2 (m, 16H, CH_2_), 1.46 (m, 4H, PCH_2_CH_2_CH_2_CH_2_), 3.6 (m, 2H, PCH_2_), 7.8–7.9 (m, 15H, Ar–H).
4. Octyl(tributyl)phosphonium bromide (PBu_3_C_8_–Br), CARSN 57702-65-5.
  Liquid.
  ^1^H NMR (400 MHz, CDCl_3_): σ = 0.86 (t, 3H, CH_3_), 0.96 (t, 9H, CH_2_CH_2_CH_2_CH_3_), 1.24 (m, 8H, CH_2_), 1.51 (m, 16H, PCH_2_CH_2_CH_2_CH_2_), 2.44 (m, 8H, PCH_2_).
5. Decyl(tributyl)phosphonium bromide (Pbu_3_C_10_–Br), CASRN 99045-50-8.
  Viscous liquid.
  ^1^H NMR (400 MHz, CDCl_3_): σ = 0.85 (t, 3H, CH_3_), 0.96 (t, 9H, CH_2_CH_2_CH_2_CH_3_), 1.24 (m, 12H, CH_2_), 1.51 (m, 16H, PCH_2_CH_2_CH_2_CH_2_), 2.43 (m, 8H, PCH_2_).
6. Dodecyl(tributyl)phosphonium bromide (Pbu_3_C_12_–Br), CASRN 15294-63-0.
  White solid, m.p.: 30–32 °C.
  ^1^H NMR (400 MHz, CDCl_3_): σ = 0.86 (t, 3H, CH_3_), 0.96 (t, 9H, CH_2_CH_2_CH_2_CH_3_), 1.24 (m, 16H, CH_2_), 1.52 (m, 16H, PCH_2_CH_2_CH_2_CH_2_), 2.44 (m, 8H, PCH_2_).

### 3.4. In Vitro Evaluation of the Activity of the PILs

#### 3.4.1. Antibacterial Activity

**Figure 1:**
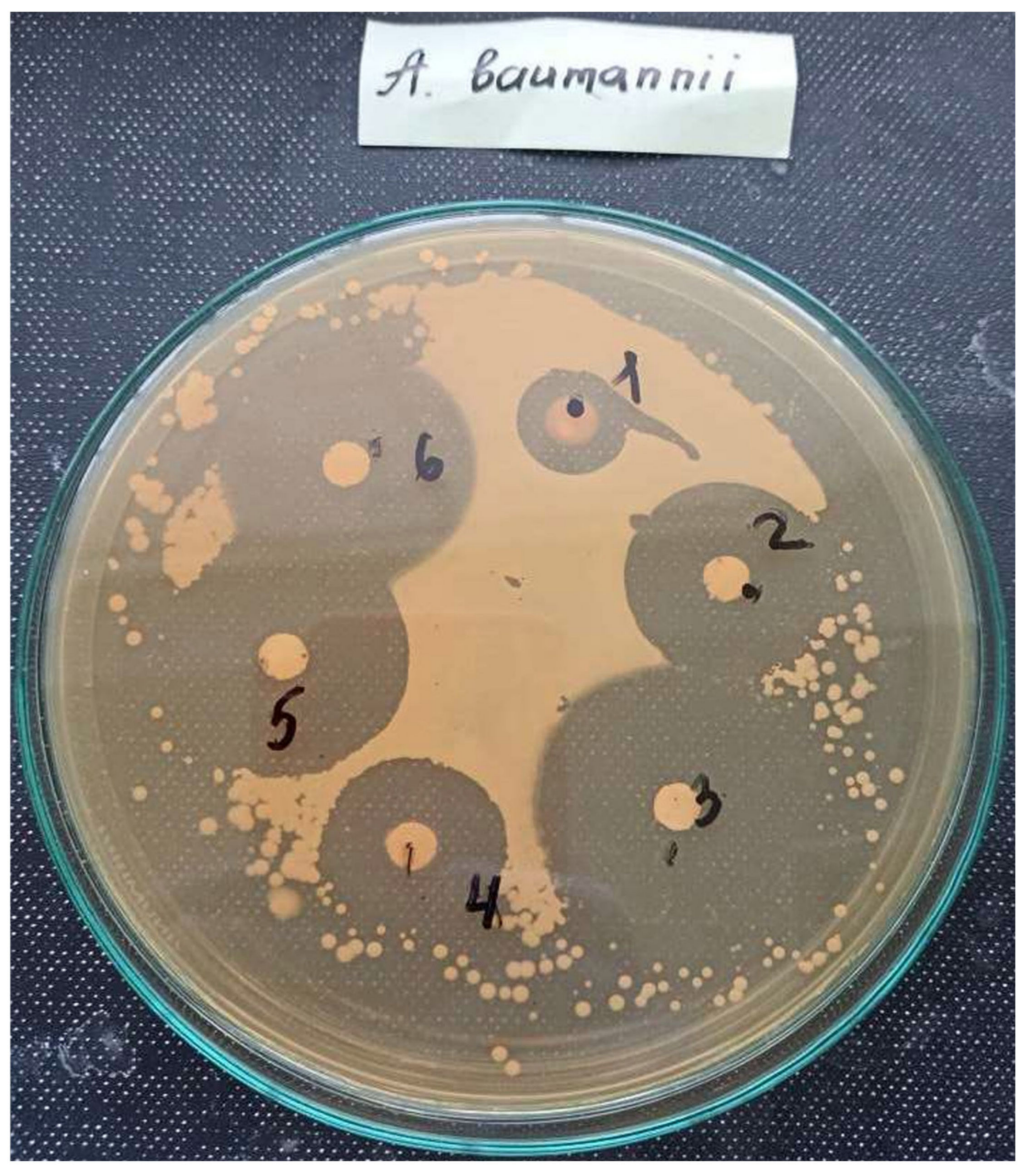
Inhibition zone diameters of the six studied PILs (content on a disk, 1.25 µmoles) of an MDR clinical isolate of *A. baumannii* on agar plates.

The biological study results of the PILs with predicted activity against MDR *A. baumannii* are presented in Table 3 and Figure 1.

**Table 3:** Inhibition zone diameters (mm) formed by the PILs against *A. baumannii* (*n* = 3).

N	Compound	Compound Content on a Disk (µmoles)			
		0.01	0.05	0.25	1.25
1	PPh_3_C_8_–Br	8.1 ± 0.3	12.3 ± 0.6	15.5 ± 0.3	17.3 ± 0.6
2	PPh_3_C_10_–Br	20.4 ± 0.9	25.4 ± 0.3	28.6 ± 0.9	31.5 ± 0.6
3	PPh_3_C_12_–Br	26.2 ± 0.6	30.1 ± 0.6	34.4 ± 0.9	41.8 ± 0.9
4	Pbu_3_C_8_–Br	14.5 ± 0.3	18.8 ± 0.3	22.3 ± 0.6	26.1 ± 0.3
5	Pbu_3_C_10_–Br	20.7 ± 0.3	25.4 ± 0.6	31.3 ± 0.3	34.2 ± 0.3
6	Pbu_3_C_12_–Br	26.1 ± 0.6	30.3 ± 0.3	33.2 ± 0.6	36.6 ± 0.6
7	Ampicillin, oxacillin, ceftriaxone	6 ± 0.3	6 ± 0.3	6 ± 0.3	6 ± 0.3

The results regarding the PILs’ antibacterial activity against MDR *A. baumannii* (Table 3 and Figure 1) demonstrated anti-*A. baumannii* activity of all the six investigated PILs in the 0.01–1.25 µmoles content on a disk. PILs dodecyl(triphenyl)phosphonium bromide (**3**), decyl(triphenyl)phosphonium bromide (**2**), dodecyl(tributyl)phosphonium bromide (**6**), and decyl(tributyl)phosphonium (**5**) bromide with the C_12_ and C_10_ alkyl chain length were found to be more active with inhibition zone diameters in the range of 31.5–41.8 mm with the content on a disk of 1.25 µmoles. PILs octyl(triphenyl)phosphonium bromide (**1**) and octyl(tributyl)phosphonium bromide (**4**) with the C_8_ alkyl chain length demonstrated a lower activity ranging from 17.3 to 26.1 mm (inhibition zone diameters) (content on a disk of 1.25 µmoles). A similar tendency was observed with decreasing µmoles on a disk to 0.01.

The MIC values were determined for PILs **2**, **4**–**6** as 12.5 µM. Furthermore, PIL **3** showed a high activity, with the MIC value of 6.25 µM, and PIL **1** had the MIC value of 25.0 µM. The obtained in vitro results confirmed the in silico prediction of antibacterial activity of the studied compounds.

#### 3.4.2. Antioxidant Activity

It is known that oxidative stress induced by microbial pathogens not only leads to disruption of the key metabolic processes in the body, but also regulates the replication of microbial pathogens and promotes the formation of bacterial resistance [53]. Antibiotics are able to regulate the production of reactive oxygen species and the bactericidal action of antimicrobial agents under aerobic conditions [54]. Thus, compounds with high antioxidant activity can be important to decrease oxidative stress and help the human body to fight bacteria.

The structural features and established anti-*A. baumannii* properties of the studied PILs formed the basis for the study of their potential antioxidant activity (AOA). The results of the AOA estimation of the test compounds by free radical lipid peroxidation are presented in Table 4.

The known synthetic antioxidant ionol (butylhydroxytoluene) was used as a reference. This compound is widely used in the production of food products (food additive E321), personal care products, cosmetics, and in some pharmaceuticals [55].

**Table 4:** Antioxidant activity of PILs (*n* = 3).

N	Compound	Inhibition Fate of MDA, %
1	PPh_3_C_8_–Br	31.5 ± 2.0
2	PPh_3_C_10_–Br	34.2 ± 1.2
3	PPh_3_C_12_–Br	33.3 ± 1.5
4	PPh_3_C_8_–Br	31.5 ± 2.0
5	PPh_3_C_10_–Br	34.2 ± 1.2
6	PBu_3_C_12_–Br	33.1 ± 2.2
	Ionol ^a^	31.4 ± 2.5
	1% DMSO ^b^	14 ± 0.5

The concentration of all the compounds was 10 mg/mL. Note: ^a^ reference antioxidant, ^b^ reference negative control. The inhibition rates indicated were reported after correction for the activity of the reference negative control (i.e., we observed 45.4% for ionol and reported a corrected value of 31.4%).

**Scheme 2:**
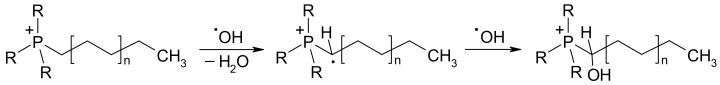
Possible mechanism of hydroxyl radical inhibition by phosphonium ILs.

Surprisingly, both alkyl triphenylphosphonium and alkyl tributylphosphonium ILs demonstrated a similar ability to inhibit lipid peroxidation processes at the ionol level ranging between 30.2% and 34.2% (Table 4). It is worth noting that the mechanism of antioxidant activity of these compounds is not described. In the case of alkyl triphenylphosphonium salts, nucleophilic radical addition of hydroxyl radicals to phenyl rings could be assumed, likely to cationic heterocyclic compounds [56]. However, aliphatic alkyl tributylphosphonium ILs also showed high antioxidant activity, similar to the triphenylphosphonium-based ones. Thus, a possible mechanism of hydroxyl radicals scavenging by phosphonium ILs may involve a break of an a-C–H bond to form an a-phosphonium intermediate which is then intercepted by another radical to create a new a-C–OH bond (Scheme 2).

## 4. Discussion

The previously created QSAR model [27] (see also Figure S1) was used to identify six anti-*A. baumannii* agents. Selected alkyl triphenylphosphonium and alkyl tributylphosphonium bromides with the C_8_, C_10_, and C_12_ alkyl chain length demonstrated a high activity against the MDR clinical isolate of *A. baumannii* as confirmed by the inhibition zone diameters of these compounds that were in the range of 33–40 mm (Table 3). These results support previous studies, which demonstrated great prospects of ILs as therapeutic agents of complex action in biomedical applications [57,58,59]. Previous studies showed that ILs with an alkyl chain length of 12 and 14 carbon atoms had the highest efficiency as antimicrobial and antitumor agents [15,16,20]. The compounds investigated in this study had the alkyl chain length of 8 and 12, and those with the C_10_ and C_12_ alkyl chain length were found to be more active against *A. baumannii* (Table 3), thus confirming the previously reported trend.

We also developed in silico models to estimate the antioxidant activity of these compounds using different machine-learning techniques of the OCHEM platform [34]. The regression models demonstrated good stability, robustness, and predictive power, as verified by cross-validation and prediction of the independent test set. The model predicted the antioxidant activity of the analyzed compounds which was further confirmed by experimental studies. Indeed, we found that all the six PILs had pronounced antioxidant properties ranging from 30% to 34%, which was similar to that of the reference antioxidant ionol.

Effective antioxidant therapy is important to optimize the treatment of infectious diseases. The possibility of using certain antioxidants as antimicrobial and antiviral agents which can block the replication of microbial pathogens is widely considered an important strategy [60,61]. A correlation between the degree of reduction of the persistent potential of resistant microorganisms under the influence of certain xenobiotics and the level of their antioxidant activity was previously established: compounds with high antioxidant activity most effectively blocked the persistent properties of microbial pathogens [62]. The use of therapeutic strategies aimed at the development of antimicrobial agents with antioxidant properties is considered one of the promising areas for imbalance-regulating redox processes caused by multidrug-resistant pathogens [63,64,65]. Biologically active compounds with a dual direction of action can lead to the successful progress of this task. Therefore, the presented in vitro and in silico analysis of the antioxidant properties of new highly active PILs make them promising pharmacologically active agents against *A. baumannii*. New theoretical and experimental studies to elucidate their mechanism of action will be important for a rational design of this class of compounds.

## 5. Conclusions

We investigated the antibacterial activity of new ionic liquids against *A. baumannii*. To study the antioxidant activity, predictive in silico models based on different machine-learning techniques were built using the OCHEM platform. The developed regression models demonstrated good stability, robustness, and predictive power, as verified by cross-validation and prediction of the test set.

Six PILs were synthesized and their anti-*A. baumannii* activity and antioxidant properties were evaluated in vitro. The studied alkyl triphenylphosphonium and alkyl tributylphosphonium bromides with the C_8_, C_10_, and C_12_ alkyl chain length demonstrated activity against the MDR clinical isolate of *A. baumannii* in the range of 17.3–41.8 mm (inhibition zone diameters) and 6.25–25.0 µM (MIC). The PILs with the C_10_ and C_12_ alkyl chain length were found to be more active, with inhibition zone diameters in the range of 31.5–41.8 mm (with MIC values in the range of 6.25–12.5 µM). The alkyl chains shorter than eight or longer than 12 carbon atoms resulted in the loss of activity of the PILs.

All the tested PILs had similar antioxidant properties at the reference antioxidant ionol level ranging from 30% to 34%. These results suggest that the investigated compounds are interesting and promising lead structures against *A. baumannii*. Additional experimental studies to elucidate their mechanism of action will be important for a further rational design of these compounds.

## Figures and Tables

**Table 2:** Chemical structures of the synthesized compounds tested for their antibacterial activity.

PILs	Compound	Molecular Weight	Chemical Structure	Chemical Name
1	PPh_3_C_8_–Br	455.41	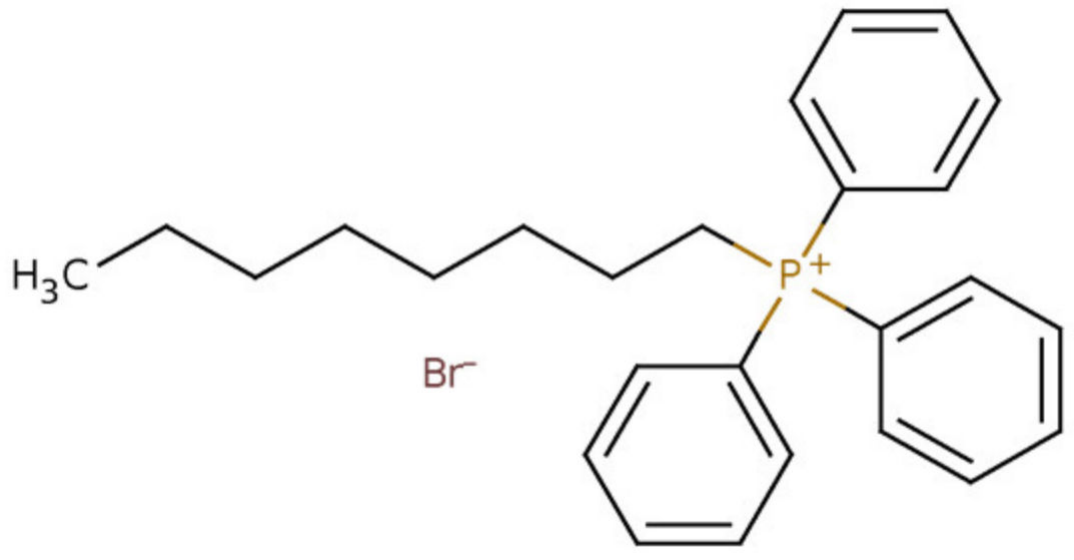	Octyl(triphenyl)phosphonium bromide
2	PPh_3_C_10_–Br	483.46	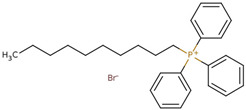	Decyl(triphenyl)phosphonium bromide
3	PPh_3_C_12_–Br	511.52	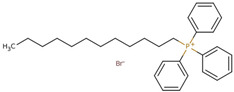	Dodecyl(triphenyl)phosphonium bromide
4	PBu_3_C_8_–Br	395.4	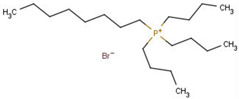	Octyl(tributyl)phosphonium bromide
5	PBu_3_C_10_–Br	423.49	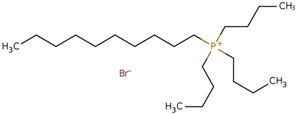	Decyl(tributyl)phosphonium bromide
6	PBu_3_C_12_–Br	451.55	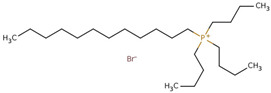	Dodecyl(tributyl)phosphonium bromide

## Data Availability

All the data and models are available at http://ochem.eu/article/139940 (accessed on 4 April 2022).

## Supplementary Materials

The following supporting information can be downloaded at https://www.mdpi.com/article/10.3390/antibiotics11040491/s1. Figure S1. Consensus classification machine-learning model [27] built by the OCHEM platform using several machine learning algorithms [66,67]. The training and test sets included 210 and 53 molecules, respectively. The cross-validation results were reported for the training set. Figure S2. QSAR models developed using OCHEM (http://ochem.eu) in this study. (**a**–**c**) Statistical coefficients calculated for the regression models by a different MLT. (**d**) Consensus model calculated by averaging the previous three models. Table S1. Anti- *A. baumannii* activity calculated by using the consensus classification model for 11 virtual compounds. Table S2. Antioxidant activity predicted by using the consensus regression model for six tested ILs. Table S3. Comparison of the melting point values of the ILs with those previously reported in the literature [68,69,70,71,72,73].

## Abbreviations

AOA	Antioxidant activity
CDK	Chemistry Development Kit
CFU	Colony-forming unit
CNF	Convolutional neural fingerprint
CNN	Convolutional neural network
ILs	Ionic liquids
MAE	Mean absolute error
MDR	Multidrug-resistant
OCHEM	Online chemical database and modeling environment
PILs	Phosphonium ionic liquids
QSAR	Quantitative structure–activity relationship
RF	Random forest
