# Supplementary material for: Theoretical and Experimental Studies of Phosphonium Ionic Liquids as Potential Antibacterials of MDR Acinetobacter baumannii
Source: Antibiotics (Basel). 2022 Apr 6;11(4):491. doi: 10.3390/antibiotics11040491 (PMC9025513; doi:10.3390/antibiotics11040491)
Supplement: Supplementary file 1 [file antibiotics-11-00491-s001.zip › antibiotics-1624615-supplementary.pdf]

Appendix

Theoretical and experimental studies of phosphonium ionic liquids as potential antibacterials of MDR *Acinetobacter baumannii*

Larysa O. Metelytsia [1], Diana M. Hodyna [1], Ivan V. Semenyuta [1],

Vasyl V. Kovalishyn [1], Sergiy P. Rogalsky [1], Kateryna Yu. Derevianko [1],

Volodymyr S. Brovarets [1], Igor V. Tetko [2,3,*]

[1] V.P. Kukhar Institute of Bioorganic Chemistry and Petrochemistry, National Academy of Science of Ukraine, 1 Murmanska Street, 02094, Kyiv-94, Ukraine; metelitsa@bpci.kiev.ua (L.O.M.); dianahodyna@gmail.com (D.M.H); ivan@bpci.kiev.ua (I.V.S.); vkovalishyn@bpci.kiev.ua (V.V.K.); sergey.rogalsky@gmail.com (S.P.R.); katerinaderevianko@gmail.com (K.Y.D.); brovarets@bpci.kiev.ua (V.S.B.)

[2] Institute of Structural Biology, Molecular Targets and Therapeutics Center, Helmholtz Munich - Deutsches Forschungszentrum für Gesundheit und Umwelt (GmbH), Neuherberg, D-85764, Germany

[3] BIGCHEM GmbH, Unterschleißheim, D-85716, Germany

* Correspondence: i.tetko@helmholtz-munich.de; Tel.: + 49-89-31873575

1. *Parameters of the Classification models*

The previously published classification models [27] were created with three machine-learning methods, including *Associative Neural Networks* (ASNN) [65], *k-Nearest Neighbors (kNNs)*, and *WEKA-RF (Random Forest)* [66] using On-line Chemical Database and Modeling Environment [34]. A consensus model was built as a simple average of the three models (http://ochem.eu/article/113921) (see Figure S1).

Model name: M4_Consensus AcinBaum_Class - 334799 [rename] , published in In silico and in vitro studies of a number PILs as new antibacterials against MDR clinical isolate Acinetobacter baumannii.
Public ID is 783

Predicted property: **AcinBaum_Class** modeled in CLASS
Training method: Consensus

Data Set	#	Accuracy	Balanced Accuracy	MCC	AUC
Training set: A_Baumanii_Set1 (training)	210 records	83% ± 3.0	82% ± 3.0	0.66 ± 0.05	0.9 ± 0.02
Test set: A_Baumanii_Set1 (test) [x]	53 records	83% ± 6.0	83% ± 5.0	0.7 ± 0.1	0.92 ± 0.04

Show ROC curves

Real↓/Predicted→	inactive	active	Hit rate
inactive	69	21	0.77
active	14	106	0.88
Precision	0.83	0.83	
Training (Original)			

Real↓/Predicted→	inactive	active	Hit rate
inactive	22	4	0.85
active	5	22	0.81
Precision	0.81	0.85	
Test (Original)			

Figure S1. Consensus classification machine learning model built by the OCHEM [34]. The training and test sets included 210 and 53 molecules, respectively. The cross-validation results were reported for the training set.

2. *Parameters of the Regression models from Table 1*

Model name: M1_AntioxidantActivity_IC50_TRANSNNI (F) (3D) _ 10/10 - 445747 [rename] , published in Theoretical phosphonium ionic liquids as potential antibacterials of MDR Acinetobacter baumannii
Public ID is 917

Predicted property: **AntioxidantActivity_IC50** modeled in -log(M)
Training method: TRANSNNI

Data Set	#	R2	q2	RMSE	MAE
Training set: Antioxid_Activity_Set_I (training)	996 records	0.77 ± 0.02	0.77 ± 0.02	0.44 ± 0.02	0.29 ± 0.01
Test set: Antioxid_Activity_Set_I (test) [x]	249 records	0.75 ± 0.05	0.75 ± 0.05	0.43 ± 0.03	0.28 ± 0.02

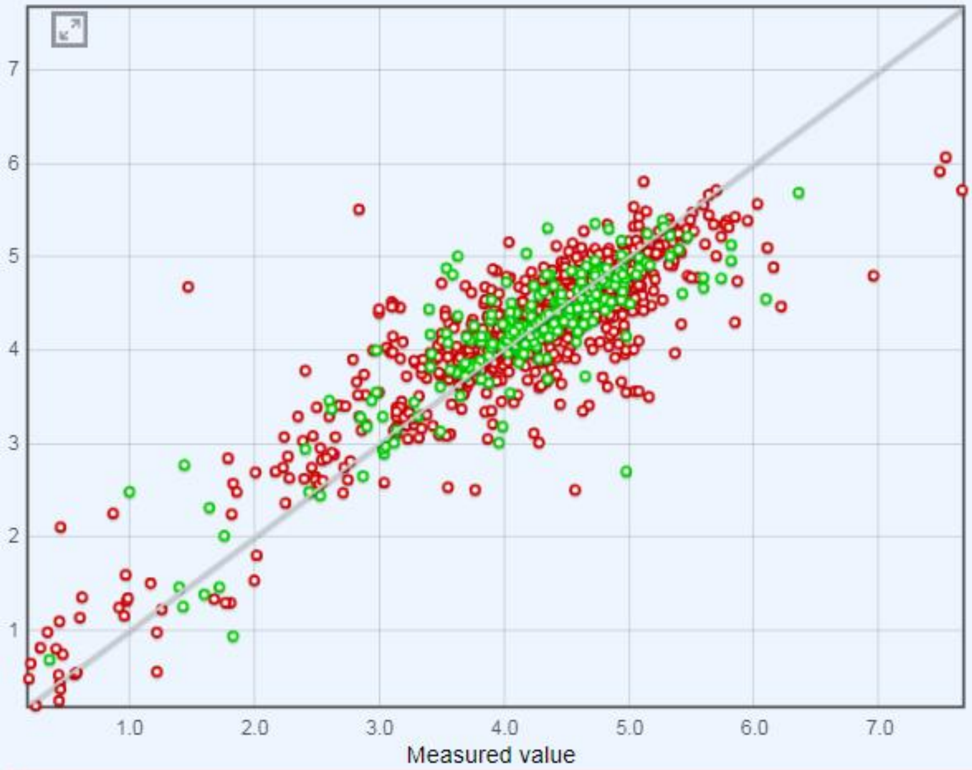

a)

Model name: M2_AntioxidantActivity_IC50_CNF2 (F) (3D) _ 10/10 - 445746 [rename] , published in Theoretical and phosphonium ionic liquids as potential antibacterials of MDR Acinetobacter baumannii
Public ID is 916

Predicted property: **AntioxidantActivity_IC50** modeled in -log(M)
Training method: CNF2

Data Set	#	R2	q2	RMSE	MAE
Training set: Antioxid_Activity_Set_I (training)	996 records	0.76 ± 0.02	0.76 ± 0.02	0.46 ± 0.02	0.3 ± 0.01
Test set: Antioxid_Activity_Set_I (test) [x]	249 records	0.77 ± 0.04	0.77 ± 0.04	0.41 ± 0.03	0.28 ± 0.02

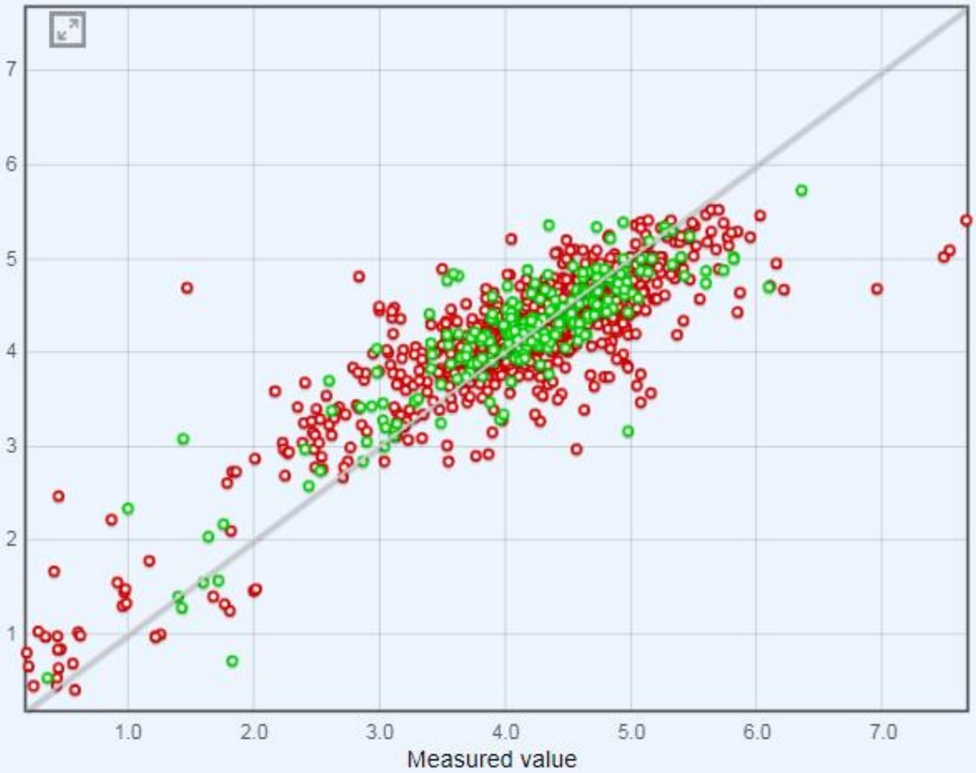

b)

Model name: M3_AntioxidantActivity_IC50_RFR _[ALogPS, CDK23 (cons,topol,geom,elect,hybr), OEstate] - 445742

Theoretical and experimental studies of phosphonium ionic liquids as potential antibacterials of MDR Acinetobacter b

Public ID is 914

Predicted property: **AntioxidantActivity_IC50** modeled in -log(M)

Training method: RFR

Data Set	#	R2	q2	RMSE	MAE
Training set: Antioxid_Activity_Set_I (training)	985 records	0.74 ± 0.03	0.73 ± 0.02	0.49 ± 0.02	0.32 ± 0.01
Test set: Antioxid_Activity_Set_I (test) [x]	249 records	0.74 ± 0.04	0.73 ± 0.04	0.44 ± 0.04	0.29 ± 0.02

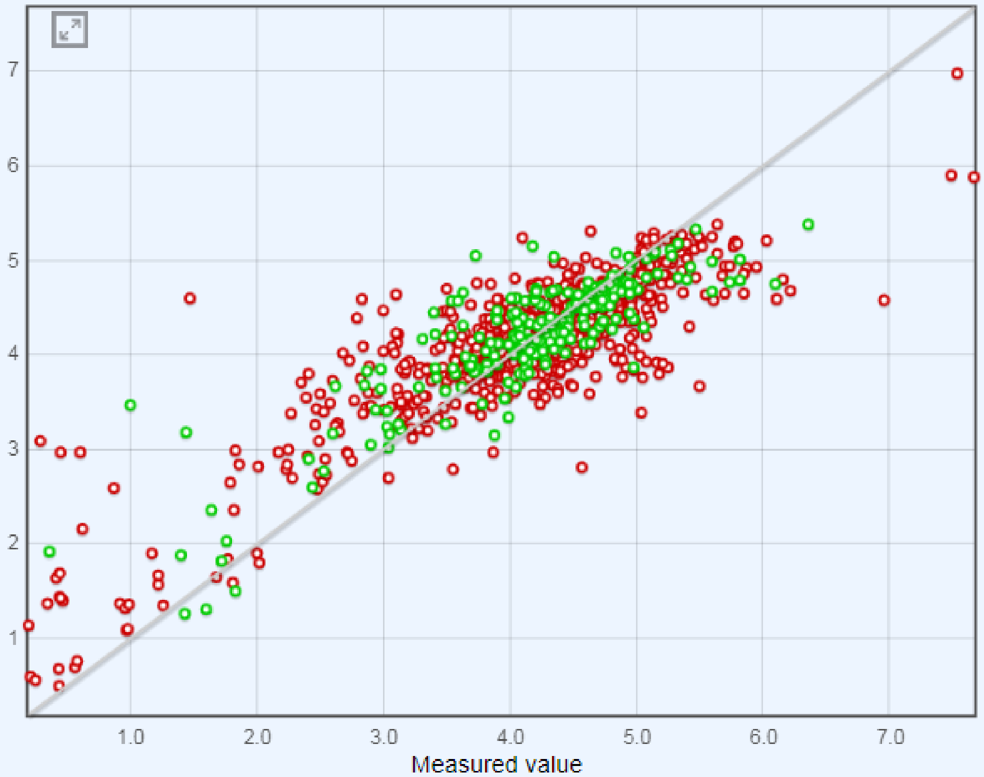

c)

Model name: M4_Consensus AntioxidantActivity_IC50 - 445890 [rename] , published in Theoretical and experimental ionic liquids as potential antibacterials of MDR Acinetobacter baumannii
Public ID is 918

Predicted property: **AntioxidantActivity_IC50** modeled in -log(M)
Training method: Consensus

Data Set	#	R2	q2	RMSE	MAE
o Training set: Antioxid_Activity_Set_I (training)	996 records	0.78 ± 0.02	0.78 ± 0.02	0.44 ± 0.02	0.29 ± 0.01
o Test set: Antioxid_Activity_Set_I (test) [x]	249 records	0.77 ± 0.04	0.77 ± 0.04	0.41 ± 0.03	0.28 ± 0.02

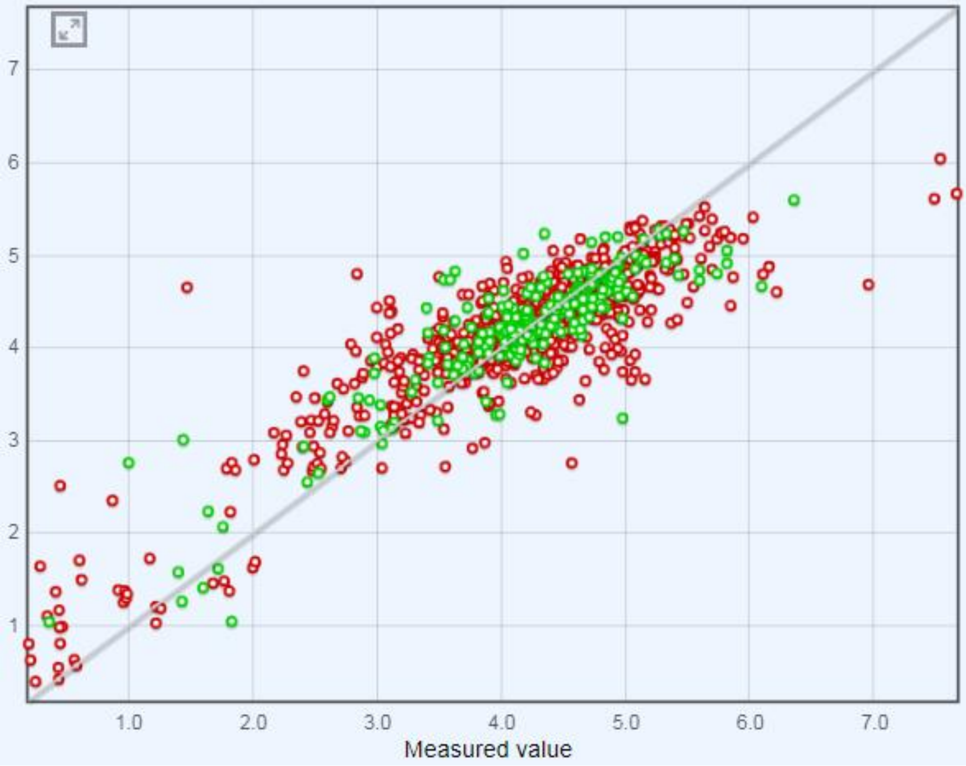

d)

Figure S2. QSAR models developed using the OCHEM (http://ochem.eu). (**a-c**) Statistical coefficients calculated for regression models by a different MLT; (**d**) Consensus model calculated by averaging the previous three models.

3. *Evaluation activity of new compounds*

Table S1. Anti-*A. baumanii* activity calculated by using the consensus classification model for 11 virtual compounds.

**Comp. No**	**SMILES**	**Predicted activity**	**CONSENSUS-STD**	**Estimated probability**	**AD**
**1***	[Br-].CCCCCCCC[P+](C1=CC=CC=C1)(C2=CC=CC=C2)C3=CC=CC=C3	active	0.34	0.62	TRUE
**2***	[Br-].CCCCCCCCCC[P+](C1=CC=CC=C1)(C2=CC=CC=C2)C3=CC=CC=C3	active	0.18	0.81	TRUE
**3***	[Br-].CCCCCCCCCCCC[P+](C1=CC=CC=C1)(C2=CC=CC=C2)C3=CC=CC=C3	active	0.18	0.81	TRUE
**4***	[Br-].CCCCCCCC[P+](CCCC)(CCCC)CCCC	active	0.09	0.76	TRUE
**5***	[Br-].CCCCCCCCCC[P+](CCCC)(CCCC)CCCC	active	0.1	0.81	TRUE
**6***	[Br-].CCCCCCCCCCCC[P+](CCCC)(CCCC)CCCC	active	0.12	0.76	TRUE
7	[O-][Cl](=O)(=O)=O.O=C(CSC1=C(N=C(S1)C1=CC=CC=C1)[P+](C1=CC=CC=C1)(C1=CC=CC=C1)C1=CC=CC=C1)C1=CC=CC=C1	inactive	0.46	0.67	FALSE
8	[Cl-].[Cl-].CC1=CC=C(C=C1)C1=NC(=C(O1)SC1=C(N=C(S1)C1=CC=CC=C1)[P+](C1=CC=CC=C1)(C1=CC=CC=C1)C1=CC=CC=C1)[P+](C1=CC=CC=C1)(C1=CC=CC=C1)C1=CC=CC=C1	inactive	0.21	0.71	TRUE
9	[O-][Cl](=O)(=O)=O.COC1=CC=C(C=C1)C1=NC(=C(O1)N1C[C@H]2C[C@H](C1)C1=CC=CC(=O)N1C2)[P+](C1=CC=CC=C1)(C1=CC=CC=C1)C1=CC=CC=C1	inactive	0.11	0.81	TRUE
10	[O-][Cl](=O)(=O)=O.ClC1=CC=C(C=C1)C1=NC(=C(O1)N1C[C@H]2C[C@H](C1)C1=CC=CC(=O)N1C2)[P+](C1=CC=CC=C1)(C1=CC=CC=C1)C1=CC=CC=C1	inactive	0.18	0.86	TRUE
11	[O-][Cl](=O)(=O)=O.FC1=CC=C(C=C1)C1=NC(=C(O1)N1C[C@H]2C[C@H](C1)C1=CC=CC(=O)N1C2)[P+](C1=CC=CC=C1)(C1=CC=CC=C1)C1=CC=CC=C1	inactive	0.17	0.81	TRUE
12	[O-][Cl](=O)(=O)=O.O=C1C=CC=C2[C@@H]3C[C@H](CN(C3)C3=C(N=C(O3)C3=CC=CC=C3)[P+](C3=CC=CC=C3)(C3=CC=CC=C3)C3=CC=CC=C3)CN12	inactive	0.18	0.81	TRUE

CONSENSUS-STD – the standard deviation of the predictions, obtained from an ensemble of models.* Compounds selected for further analysis. * Estimated probability of the predicted activity class based on consensus model [34].

Table S2. Antioxidant activity prediction by using the consensus regression model for six tested ILs.

Comp. No	IC_{50}, µg/ml	log(1/IC_{50}), M	CONSENSUS-STD	RMSE	AD
1	46.6	3.99	0.08	0.35	TRUE
2	55.5	3.94	0.06	0.35	TRUE
3	64.4	3.9	0.06	0.35	TRUE
4	33.7	4.07	0.11	0.35	TRUE
5	35.2	4.08	0.06	0.35	TRUE
6	45.2	3.99	0.08	0.35	TRUE

CONSENSUS-STD – the standard deviation of the predictions, obtained from an ensemble of models. IC_{50} value of DPPH radical scavenging activity (DPPH- 2,2-diphenyl-1-picryl-hydrazyl), RMSE – predicted root mean square error; AD – applicability domain.

Table S3. Comparison of melting point values of ILs with those previously reported in the literature.

n	Compound	CASRN	MW	Chemical Name	MP this work	MP published	Ref.
1	PPh_3C_8-Br	42036-78-2	455.41	Octyltriphenylphosphonium bromide	60-62 °C	61-63°C<br>66-71°C	[67]<br>[68]
2	PPh_3C_{10}-Br	32339-43-8	483.46	Decyltriphenylphosphonium bromide	86-88 °C	90°C	[69]
3	PPh_3C_{12}-Br	15510-55-1	511.52	Dodecyltriphenylphosphonium bromide	89-92 °C	93.0-93.5<br>88-95 °C	[70]<br>[68]
4	PBu_3C_8-Br	57702-65-5	395.4	Octyltributyl phosphonium bromide	liquid	n.a.	
5	PBu_3C_{10}-Br	99045-50-8	423.49	Decyltributylphosphonium bromide	viscous liquid	n.a.	
6	PBu_3C_{12}-Br	15294-63-0	451.55	Dodecyltributylphosphonium bromide	30-32°C	33°C<br>33°C	[71]<br>[72]

References

27. Trush, M.M.; Kovalishyn, V.; Hodyna, D.; Golovchenko, O.V.; Chumachenko, S.; Tetko, I.V.; Brovarets, V.S.; Metelytsia, L. In Silico and in Vitro Studies of a Number PILs as New Antibacterials against MDR Clinical Isolate Acinetobacter Baumannii. *Chem. Biol. Drug Des.* **2020**, *95*, 624–630, doi:10.1111/cbdd.13678.
34. Sushko, I.; Novotarskyi, S.; Körner, R.; Pandey, A.K.; Rupp, M.; Teetz, W.; Brandmaier, S.; Abdelaziz, A.; Prokopenko, V.V.; Tanchuk, V.Y.; et al. Online Chemical Modeling Environment (OCHEM): Web Platform for Data Storage, Model Development and Publishing of Chemical Information. *J. Comput. Aided Mol. Des.* **2011**, *25*, 533–554, doi:10.1007/s10822-011-9440-2.
65. Tetko, I.V. Associative Neural Network. *Methods Mol. Biol. Clifton NJ* **2008**, *458*, 185–202.
66. Frank, E.; Hall, M.; Trigg, L.; Holmes, G.; Witten, I.H. Data Mining in Bioinformatics Using Weka. *Bioinformatics* **2004**, *20*, 2479–2481, doi:10.1093/bioinformatics/bth261.
67. N-Octyl Triphenylphosphonium Bromide Available online: https://www.chemsrc.com/en/cas/42036-78-2_799327.html (accessed on 18 February 2022).
68. Kuz'menok, N.M.; Mikhalyonok, S.G.; Arol, A.S.; Shevchuk, M.O.; Bezborodov, V.S.; Krakhalev, M.N.; Sutormin, V.S.; Prishchepa, O.O.; Zharkova, G.M.; Zyryanov, V.Y. Synthesis of Organotriphenylphosphonium Halides, Quaternary Ammonium Salts and Study of Their Application as Surfactants Soluble in Liquid Crystals. *Zhidkie Krist. Ikh Prakt. Ispolzovanie* **2020**, *20*, 6–18, doi:10.18083/LCAppl.2020.1.6.
69. Decyl-TPP Available online: https://www.chemsrc.com/en/cas/32339-43-8_255481.html (accessed on 18 February 2022).
70. Ivashchenko, S. P. Lipids. XLIV. A New Synthesis of 1-Alken-1-Yl Alkyl Ethers Using the Wittig Reaction. *Zhurnal Organicheskoi Khimii* **1966**, *2*, 2181–2183.
71. Kurt, M. Antiseptic Detergent Compositions. Avaialble online: https://patents.google.com/patent/US3281365A (accessed on 4 April 2022).
72. Tributyl(Dodecyl)Phosphonium Bromide Available online: https://www.chemsrc.com/en/cas/15294-63-0_1102483.html (accessed on 19 February 2022).
